# Characterizing Patients with Uncontrolled Blood Pressure at an Urban Hospital in Hanoi, Vietnam

**DOI:** 10.1155/2020/5710281

**Published:** 2020-09-10

**Authors:** Hoang Thanh Nguyen, Nam Hoang Thi Phuong, Ngoc Tran Nguyen, Tuan Nguyen Anh, Vung Nguyen Dang

**Affiliations:** ^1^Hanoi Medical University, Hanoi, Vietnam; ^2^National Geriatric Hospital, Hanoi, Vietnam; ^3^National Institute of Mental Health, Bach Mai Hospital, Hanoi, Vietnam; ^4^Viet Duc University Hospital, Hanoi, Vietnam

## Abstract

Great efforts to advance the diagnosis and treatment of hypertension for controlling hypertension have been made; however, the rates of uncontrolled blood pressure are still high. This study explored the rate of uncontrolled hypertension in patients with hypertension managed in an urban hospital of Vietnam and identified associated factors. A cross-sectional survey was performed from August to October 2019 among hypertensive patients at an urban hospital in Hanoi, Vietnam. Blood pressure was evaluated at the time of medical examination. Demographic, clinical, and behavioral characteristics were also collected. Multivariate logistic regression was used to identify the factors related to uncontrolled hypertension. Among 220 patients, the rate of uncontrolled hypertension was 40.5%. Females had a lower likelihood of having uncontrolled hypertension compared to males (adjusted OR = 0.33; 95% CI = 0.11–0.98). Higher duration of diseases (adjusted OR = 1.07; 95% CI = 1.01–1.14) and higher body mass index (adjusted OR = 1.23; 95% CI = 1.05–1.45) were positively associated with uncontrolled hypertension. Patients who carried supplies needed for self-care, cut down on stress, exercised regularly, and stopped/cut down on smoking were also less likely to develop uncontrolled hypertension. This study reveals that uncontrolled hypertension was common among hypertensive patients in Vietnam. Improving self-care capacity and encouraging healthy behaviors are critically important to control blood pressure, particularly among patients who were males and had high disease duration and body mass index.

## 1. Introduction

High blood pressure, or hypertension, has been well recorded as a leading cause of morbidity and mortality in both developed and developing nations. People with hypertension are particularly vulnerable to cardiovascular diseases (CVDs), stroke, or renal failures [1]. It is estimated that the global prevalence of hypertension in adults is 30.8% [2]. Great efforts to advance the diagnosis and treatment of hypertension for controlling hypertension have been made; however, the rates of uncontrolled blood pressure are still high [1, 3, 4], especially in Asian countries. Prior literature in China and Japan indicated that among hypertensive patients receiving treatment, uncontrolled hypertension was observed in 62.5% and 62.9% of hypertensive patients, respectively [2, 5], while these rates in the United States (US) and the United Kingdom (UK) were 31.1% and 39.2%, correspondingly [2]. A report from the World Health Organization revealed that more than one billion people are experiencing uncontrolled hypertension worldwide [6]. Many factors have been found that are attributed to this condition, consisting of clinical (e.g., gender, age, duration of diseases, comorbidities, and nonadherence to medication) and behavioral factors (e.g., unhealthy and sedentary lifestyles) [7–9]. However, these risk factors are varied and inconsistent among studies. Given the severe consequences of uncontrolled hypertension [10, 11], attempts to identify risk factors for this condition are important to make it understandable, which helps to design appropriate management strategies in clinical settings.

Vietnam has been witnessed a rapid epidemiological transition in the last decade, resulting in a significant increase in noncommunicable diseases, including hypertension [12]. A recent meta-analysis figured out that 21.1% of Vietnamese adults are living with hypertension [13]. To date, only one study performed in the community found that 37.7% of patients who received antihypertensive medication developed uncontrolled blood pressure [14]. However, studies to investigate thoroughly the determinants of uncontrolled blood pressure in hospital-based settings are insufficient. This study explored the rate of uncontrolled hypertension in patients with hypertension managed in an urban hospital of Vietnam and identified associated factors.

## 2. Materials and Methods

### 2.1. Study Design and Sampling Method

A cross-sectional survey was performed from August to October 2019 among hypertensive patients at an urban hospital in Hanoi, Vietnam. This hospital currently managed approximately two thousand hypertensive patients living and working in Hanoi, Vietnam. Participants were eligible for the study if they had been confirmedly diagnosed to suffer from hypertension according to the guideline of Vietnam Ministry of Health (persistently systolic blood pressure level of ≥140 mmHg and/or persistently diastolic blood pressure level of ≥ 90 mmHg). Moreover, participants had to be at least 18 years old, registered to manage and treat hypertension in the hospital. We excluded patients who (1) had impaired cognitive conditions and (2) were inpatients or hypertensive patients without registration of chronic disease management at the hospital. Among 250 patients who were invited to participate in the study, a total of 220 patients (response rate 88%) agreed to be enrolled in the survey.

### 2.2. Data Collection and Measurement

Data collection was carried out by undergraduate medical students from Hanoi Medical University. They were trained carefully in communication skills with patients as well as manners to collect data consistently. Patients were invited when they finished all procedures (e.g., examining medical conditions, having a blood test, and receiving drug prescription) and waited for medication dispense. If they were willing to participate, they were invited to a private counseling room for an interview and to protect their privacy. Patients have informed briefly the purpose of the study as well as their benefits. After completing the survey, their weight and height were measured.

#### 2.2.1. Primary Outcome

Office blood pressure was evaluated by using the Japanese Alpk2 sphygmomanometer at the time of medical examination. Blood pressure was measured twice; the second measure was done 10 minutes after the first measure. The mean of the two measures was used for analysis. Uncontrolled hypertensive patients were defined as those having a systolic blood pressure level of ≥140 mmHg and/or diastolic blood pressure level of ≥90 mmHg according to the guideline of Vietnam Ministry of Health; otherwise, they were classified as controlled hypertensive patients.

#### 2.2.2. Covariates

During the interview, patients were asked to report their sociodemographic and behavioral characteristics (e.g., education, occupation, living area, smoking status, and alcohol drinking). Weight, height, and body mass index were measured after completing the survey. Other demographic and clinical covariates such as age, gender, comorbidities, number of antihypertensives used, last low-density lipoprotein (LDL), last high-density lipoprotein (HDL), and last triglyceride measures were extracted from the electronic medical record system of the hospital. We also employed eight items from the Condition-specific Recommendations and Adherence scale to evaluate the frequency of different recommended health behaviors for hypertensive patients [15]. These behaviors included the following: (1) take prescribed medication daily, (2) follow a low-salt diet, (3) follow a low-fat diet, (4) exercise regularly, (5) stop/cut down on smoking, (6) cut down on alcohol, (7) cut down on stress, and (8) carry supplies needed for self-care. Each behavior has six levels of response about frequency from 0 “none of the time” to 5 “all of the time”. Those answering options 0–2 were classified into the “Nonadherence” group while other patients were identified as the “Adherence” group. The total score of this scale ranges from 0 to 40, in which patients having 32/40 points were categorized as “overall health behavior adherence”; otherwise, they were categorized into “overall nonadherence” group [15].

### 2.3. Statistical Analysis

Descriptive statistics and multivariable regression models were applied in this study. Since continuous variables in this study, namely, age, duration of disease, number of comorbidities, body mass index, last low-density lipoprotein, last high-density lipoprotein, last triglyceride, and adherence score had nonnormal distribution, median and interquartile range were presented. Chi-squared and Mann–Whitney tests were used to examine the difference of demographic, clinical, and behavioral characteristics between controlled and uncontrolled hypertensive patients. Multivariate logistic regression, along with a stepwise forward selection strategy, was employed to identify associated factors with hypertensive conditions. The log-likelihood test was performed with a threshold of a *p* value of less than 0.2 to select the variables. Adjusted odds ratios (OR), *p* value, and 95% confidence interval (CI) were revealed. The level of statistical significance was set at the 5% level.

## 3. Results and Discussion

Among 220 patients in our sample, the rate of uncontrolled hypertension was 40.5%. This condition was found to be significantly higher in male patients (48.2%) than in female one (33.0%) (*p* < 0.05). Meanwhile, there was no difference in the rate of uncontrolled hypertension among age, education, occupation, living location, smoking, and alcohol groups (*p* > 0.05) (Table 1).

Table 2 shows the clinical characteristics of the sample. The highest proportion of patients had experienced hypertension for more than five years (47.7%) and used three medications (35.0%). No significant difference was found between controlled and uncontrolled hypertension groups regarding the duration of diseases, number of medications, overweight/obesity status, number of comorbidities, last LDL/HDL, and last triglyceride. Uncontrolled hypertensive patients had significantly higher body mass index (median = 24.0, IQR = [22.3–26.1]) than controlled hypertensive group (median = 23.4, IQR = [21.8–25.2]) (*p* < 0.05).

Meanwhile, none of the behavior was found to be significantly different between controlled and uncontrolled hypertension groups except “carrying supplies needed for self-care”. The rate of uncontrolled hypertension among patients who did not carry these supplies frequently (45.4%) was remarkably higher than their counterparts (31.7%) (*p* < 0.05) (Table 3).

The results of multivariate logistic regression are presented in Table 4. Females had a lower likelihood of having uncontrolled hypertension compared to males (OR = 0.33; 95% CI = 0.11–0.98). Higher duration of diseases (OR = 1.07; 95% CI = 1.01–1.14) and higher body mass index (OR = 1.23; 95% CI = 1.05–1.45) were positively associated with uncontrolled hypertension. Patients who carried supplies needed for self-care, cut down on stress, exercised regularly, and stopped/cut down on smoking were also less likely to develop uncontrolled hypertension.

## 4. Discussion

The present study revealed the substantially high rate of uncontrolled hypertension (40.5%) among patients receiving antihypertensive treatment in a Vietnamese hospital. Moreover, risk factors for uncontrolled hypertensive conditions were found comprising being male, increasing body mass index, following unhealthy or sedentary lifestyles, and not preparing essential medications for self-care. This study indicated critical results that can suggest important implications for controlling hypertension in hospital settings of Vietnam.

The rate of uncontrolled hypertension among hypertensive patients in our study (40.5%) could be comparable to the previous study performed in ten provinces of Vietnam (37.7%) [14]. This finding was lower compared to other countries in Asia and Africa such as China (62.5%), Japan (62.9%) [2, 5], India (63.6%) [16], South Africa (75.5%) [17], Democratic Republic of the Congo (77.5%) [18], Ghana (57.7%) [19], and Ethiopia (52.5%) [20], but higher than that in Thailand (24.6%) [1], the USA (31.1%), and the UK (39.2%) [2]. The differences in demographic and clinical characteristics of hypertensive patients across studies regarding gender, medication adherence, body mass index, comorbidity, and behaviors (e.g., alcohol use or smoking) might be the reasons for the diversity of uncontrolled hypertension prevalence. Nonetheless, approximately half of hypertensive patients had uncontrolled blood pressure suggesting urgent needs of comprehensive management strategies to address this issue and improve patients' health outcomes.

Male patients tended to be at higher risk of uncontrolled hypertension, which was supported by several studies in both developed and developing countries [21–23]. Literature indicated that lower concentrations of prorenin and renin contributed to the lower blood pressure among females compared to males [24, 25]. In addition, having a higher disease duration was associated with a higher risk of uncontrolled hypertension. We supposed that these patients also had a higher age, and this factor was found to be related to uncontrolled hypertension in other prior studies [21, 26]. Nonadherence medication might not be a reasonable cause of this phenomenon since almost all patients complied with the prescribed regimes. Therefore, it is a great challenge to determine an effective approach for controlling blood pressure in these populations. These patients should also be prioritized for further interventions to reduce the burden of hypertension.

Our study was in line with prior research that increasing body mass index was a major risk factor for uncontrolled hypertension [19, 27–29]. Indeed, the dose-response relationship between body mass index and blood pressure has been fully investigated [4, 30]. Overweight or obesity could activate the sympathetic nervous and renin-angiotensin systems as well as increase the reabsorption of kidneys via sodium retention, leading to the development of obesity-related high blood pressure [4, 30]. Therefore, weight reduction has been proposed widely which is an effective intervention to control the blood pressure [31]. Doing physical activity or exercising regularly, thus, plays an important role in this strategy. This behavior not only reduces the bodyweight but also improves the renal function and nervous system performance as well as enhances vasoconstriction regulation [32]. Our regression model confirmed this association, showing that patients doing physical exercise frequently had a lower likelihood of having uncontrolled hypertension. Additionally, the findings of this study also underlined the benefits of cutting down smoking and stress to the improvement of blood pressure in hypertensive patients [33–35]. A prior systematic review concluded that chronic stress could increase the risk of hypertension and uncontrolled hypertension [36]. Notably, encouraging patients to prepare and carry supplies needed for hypertension when going out could reduce the risk of uncontrolled hypertension. This behavior reflects the ability for self-care in patients, which is a vital component for effective blood pressure management [19, 37].

Several major limitations should be acknowledged. First, data from this study were obtained via a cross-sectional design, which did not allow us to measure the causal relationship between uncontrolled hypertension among hypertensive patients and its related factors. Second, our sample was recruited by using a convenient sampling method in one hospital; therefore, the result of this study should be cautious about being applied in other settings. Third, several variables such as home blood pressure monitoring, class of drugs, or clinical inertia therapeutic were not included in this study. Further research should be warranted to investigate associations between these factors and uncontrolled blood pressure.

## 5. Conclusions

This study reveals that uncontrolled hypertension was common among hypertensive patients in Vietnam. Improving self-care capacity and encouraging healthy behaviors are critically important to control blood pressure, particularly among patients who were males and had high disease duration and body mass index.

## Figures and Tables

**Table 1:** Sociodemographic characteristics of hypertensive patients.

Characteristics	Controlled hypertension		Uncontrolled hypertension		Total		*p*
	*n*	%	*n*	%	*n*	%	
Total	131	59.6	89	40.5	220	100.0	
Age group							
<60 years	39	59.1	27	40.9	66	30.0	0.99
60–69 years	56	60.2	37	39.8	93	42.3	
≥70 years	36	59.0	25	41.0	61	27.7	
Gender							
Male	56	51.9	52	48.2	108	49.1	0.02
Female	75	67.0	37	33.0	112	50.9	
Education							
< High school	55	56.7	42	43.3	97	44.3	0.67
High school	32	64.0	18	36.0	50	22.8	
> High school	44	61.1	28	38.9	72	32.9	
Occupation							
Self-employed	58	59.2	40	40.8	98	46.0	0.69
Retired	56	62.9	33	37.1	89	41.8	
Others	14	53.9	12	46.2	26	12.2	
Living location							
Rural	108	57.8	79	42.3	187	85.4	0.24
Urban	22	68.8	10	31.3	32	14.6	
Current smokers							
No	114	59.1	79	40.9	193	87.7	0.70
Yes	17	63.0	10	37.0	27	12.3	
Alcohol drinkers							
No	97	61.8	60	38.2	157	71.4	0.29
Yes	34	54.0	29	46.0	63	28.6	

**Table 2:** Clinical characteristics of hypertensive patients.

Characteristics	Controlled hypertension		Uncontrolled hypertension		Total		*p*
	*n*	%	*n*	%	*n*	%	
*Comorbidities*							
Cardiac diseases	39	63.9	22	36.1	61	27.7	0.41
Stroke	5	71.4	2	28.6	7	3.2	0.52
Cerebrovascular diseases	7	70.0	3	30.0	10	4.6	0.49
Vascular diseases	19	76.0	6	24.0	25	11.4	0.08
Kidney diseases/failure	9	52.9	8	47.1	17	7.7	0.56
Metabolic syndrome	12	38.7	19	61.3	31	14.1	0.01
Blood lipid disorders	56	62.2	34	37.8	90	40.9	0.50
Digestive diseases	28	77.8	8	22.2	36	16.4	0.02
Diabetes	26	53.1	23	46.9	49	22.3	0.29
Cancer	1	50.0	1	50.0	2	0.9	0.78
Spine/joint pain	57	64.0	32	36.0	89	40.5	0.26
Liver diseases	9	40.9	13	59.1	22	10.0	0.06
Prostate diseases	7	70.0	3	30.0	10	4.6	0.49
Vestibular diseases	5	83.3	1	16.7	6	2.7	0.23

*Duration of disease (years)*							
1– < 2 years	29	56.9	22	43.1	51	23.2	0.50
2–5 years	42	65.6	22	34.4	64	29.1	
>5 years	60	57.1	45	42.9	105	47.7	
Number of antihypertensive drugs used							
1	21	61.8	13	38.2	34	15.5	0.77
≥2	110	59.1	76	40.9	186	84.6	

*Overweight/obesity*							
No	59	64.8	32	35.2	91	41.4	0.18
Yes	72	55.8	57	44.2	129	58.6	
	Median	IQR	Median	IQR	Median	IQR	*p*
Duration of disease (years)	5	[3–10]	6	[2.2–10]	5	[3–10]	0.56
Number of comorbidities	2	[1–3]	2	[1–3]	2	[1–3]	0.72
Body mass index (kg/m^2^)	23.4	[21.8–25.2]	24.0	[22.3–26.1]	23.7	[21.9–25.4]	0.02
Last low-density lipoprotein (LDL, mmol/L)	2.5	[1.4–3.3]	2.3	[1.3–3.2]	2.5	[1.4–3.3]	0.67
Last high-density lipoprotein (HDL, mmol/L)	1.3	[1.1–1.6]	1.2	[1.1–1.4]	1.2	[1.1–1.6]	0.18
Last triglyceride (mmol/L)	1.8	[1.4–2.4]	2.0	[1.3–2.9]	1.9	[1.4–2.8]	0.35

**Table 3:** Health behavior adherence of hypertensive patients.

Health behavior adherence	Controlled hypertension		Uncontrolled hypertension		Total		*p*
	*n*	%	*n*	%	*n*	%	
Take prescribed medication daily							
No	0	0.0	1	100.0	1	0.4	0.22
Yes	131	59.8	88	40.2	219	99.6	
Follow a low-salt diet							
No	68	61.8	42	38.2	110	50.5	0.42
Yes	61	56.5	47	43.5	108	49.5	
Follow a low-fat diet							
No	67	63.8	38	36.2	105	47.7	0.22
Yes	64	55.7	51	44.4	115	52.3	
Exercise regularly							
No	17	51.5	16	48.5	33	15.0	0.31
Yes	114	61.0	73	39.0	187	85.0	
Stop/cut down on smoking							
No	30	55.6	24	44.4	54	24.6	0.49
Yes	101	60.8	65	39.2	166	75.5	
Cut down on alcohol							
No	24	58.5	17	41.5	41	18.6	0.88
Yes	107	59.8	72	40.2	179	81.4	
Cut down on stress							
No	52	56.5	40	43.5	92	42.0	0.47
Yes	78	61.4	49	38.6	127	58.0	
Carry supplies needed for self-care							
No	77	54.6	64	45.4	141	64.1	0.04
Yes	54	68.4	25	31.7	79	35.9	
Overall health behavior adherence							
No	91	60.3	60	39.7	151	69.6	0.56
Yes	37	56.1	29	43.9	66	30.4	

	Median	IQR	Median	IQR	Median	IQR	
Adherence score	29	[24–33]	28	[24–32]	29	[24–33]	0.55

**Table 4:** Multivariate logistic regression to identify associated factors with uncontrolled hypertension.

	Adjusted OR		*p* value	95% confidence interval
Gender				
Male	REF			
Female	0.33	0.04	0.11	0.98
Using alcohol				
No	REF			
Yes	0.39	0.14	0.11	1.36
Carry supplies needed for self-care				
No	REF			
Yes	0.33	0.03	0.12	0.90
Follow a low-fat diet				
No	REF			
Yes	1.94	0.16	0.77	4.86
Cut down on stress				
No	REF			
Yes	0.35	0.03	0.14	0.89
Exercise regularly				
No	REF			
Yes	0.23	0.03	0.06	0.87
Stop/cut down on smoking				
No	REF			
Yes	0.21	0.01	0.06	0.70
Overall health behavior adherence				
No	REF			
Yes	3.02	0.07	0.92	9.88
Duration of disease (years)	1.07	0.03	1.01	1.14
Body mass index (kg/m^2^)	1.23	0.01	1.05	1.45
Last high-density lipoprotein (HDL, mmol/L)	0.73	0.06	0.53	1.02
Last triglyceride (mmol/L)	1.37	0.06	0.99	1.88

## Data Availability

The data used to support the findings of the study are available from the corresponding author upon request.
